# First evidence of schmallenberg virus infection in southern Italy

**DOI:** 10.1186/s12917-023-03666-5

**Published:** 2023-07-28

**Authors:** Gianmarco Ferrara, Kerstin Wernike, Giuseppe Iovane, Ugo Pagnini, Serena Montagnaro

**Affiliations:** 1 Department of Veterinary Medicine and Animal Productions, University of Naples Federico II, Via Federico Delpino n.1, Naples, 80137 Italy; 2 Friedrich-Loeffler-Institut, Südufer 10, 17493 Greifswald-Insel Riems, Germany

**Keywords:** Schmallenberg virus, Simbu serogroup, Seroprevalence, Water Buffalo, Ruminants, Italy, Bunyavirus

## Abstract

**Background:**

Schmallenberg virus (SBV) is a vector-borne pathogen that mainly affects ruminants. Schmallenberg disease has never been described in southern Italy, although this geographic area displays climatic features suitable for *Culicoides* biting midges, which transmit the pathogen. An observational study was carried out in the Campania region in 2020 to evaluate the seroprevalence in cattle and water buffalo as well as to identify the risk factors involved in the distribution of SBV.

**Results:**

Relatively high seroprevalences of 38.2% (cattle) and 43% (water buffalo) were found by using a commercial SBV ELISA, which is comparable to the prevalence obtained in other countries under post-epidemic conditions. A virus neutralization assay performed on positive samples showed high titers in a large percentage of animals which is assumed to indicate recent exposure. Bivariate analysis of several variables revealed some environmental factors associated with higher seroprevalence, such as mean annual temperature, distance from the coast, and altitude. Multivariate logistic regression confirmed the statistical association only for mean annual temperature, that was found to be the main factor responsible for the distribution of the virus in southern Italy. In addition, molecular diagnosis attempts were performed on serum samples and resulted in the detection of SBV RNA in two herds and six animals.

**Conclusions:**

In this work we have demonstrated the circulation of SBV in southern Italy using both molecular and serological assays. This study emphasized the essential role of monitoring in preventing the re-emergence of vector-borne diseases in ruminants.

**Supplementary Information:**

The online version contains supplementary material available at 10.1186/s12917-023-03666-5.

## Background

Schmallenberg virus (SBV) is an enveloped virus belonging to the Simbu serogroup, genus *Orthobunyavirus*, in the family *Bunyaviridae* [1]. This pathogen was first described in the fall of 2011 in northwestern Germany, where fever, premature births, stillbirths, and severe malformations were reported in newborn ruminants [2, 3]. Like other Simbu serogroup viruses, SBV is an arbovirus, meaning that it is mainly transmitted through the bite of hematophagous insect vectors, especially *Culicoides* spp., thanks to which it spread rapidly throughout Europe [4, 5]. In 2012, a report from the European Food Safety Agency (EFSA) showed that the virus had been reported in 14 Central European countries, and a few months later, the virus was described in Eastern Europe. Nowadays, SBV is considered endemic in Europe, and occasional outbreaks and virus circulation have been documented in several countries, including Mediterranean countries (such as Italy, Spain and Greece) and Northern Europe [6].

Due to its teratogenic potential and decrease in milk production, SBV results in economic losses and has a negative impact on the livestock industry [2]. Infected animals may exhibit non-specific clinical signs, including fever, decreased milk yield and diarrhea, but when infection occurs in naïve pregnant females it may result in reproductive disorders, and congenital malformations (especially the arthrogryposis–hydranencephaly complex) [7]. Although the impact of the infection on the dairy industry is thought to be low, some countries have imposed commercial restrictions (mainly on embryos and semen) on non-free countries, which can only be overcome through health attestations [8]. Although effective vaccines have been produced, they have never been widely implemented [9]. The decline in herd immunity mainly due to replacement of animals by naïve youngstock, combined with environmental conditions favorable to vectors, may open the door to the re-emergence of SBV and regular large-scale outbreaks in Europe [6]. This is also confirmed for other bunyavirus widespread in Asia and Australia such as Akabane virus and Aino virus, that re-emerge when the condition allows [10–12]. The same has been demonstrated for bluetongue virus, an orbivirus that shares several aspects in epidemiology with SBV such as the affected mammalian host species as well as the insect vector [5]. Since re-emergence of SBV could occur in any European country, continuous surveillance against this virus is required in order to implement sanitary measures that will contain the disease.

SBV or specific antibodies have been described in Europe in the main domestic ruminant species bred in these countries as well as in several wild ruminant and non-ruminant species (including wild boar) [13–20]. Evidence of virus circulation has been described in Italy, where SBV first appeared in February 2012 (detected in the tissues of a goat fetus in the Veneto region) [21–23]. Since then, the disease has never been reported despite the repeated outbreaks in other European states and the presence of the vector insect throughout the territory of Italy [21, 24, 25]. Furthermore, the virus has not been detected in southern Italy, and the disease has never been described in the Mediterranean buffalo, which is extensively raised in the country’s south.

The purpose of this study was to explore the SBV circulation in the Campania region (southern Italy), using serological and molecular approaches, as well as to identify possible risk factors involved in SBV distribution.

## Results

**Fig. 1:**
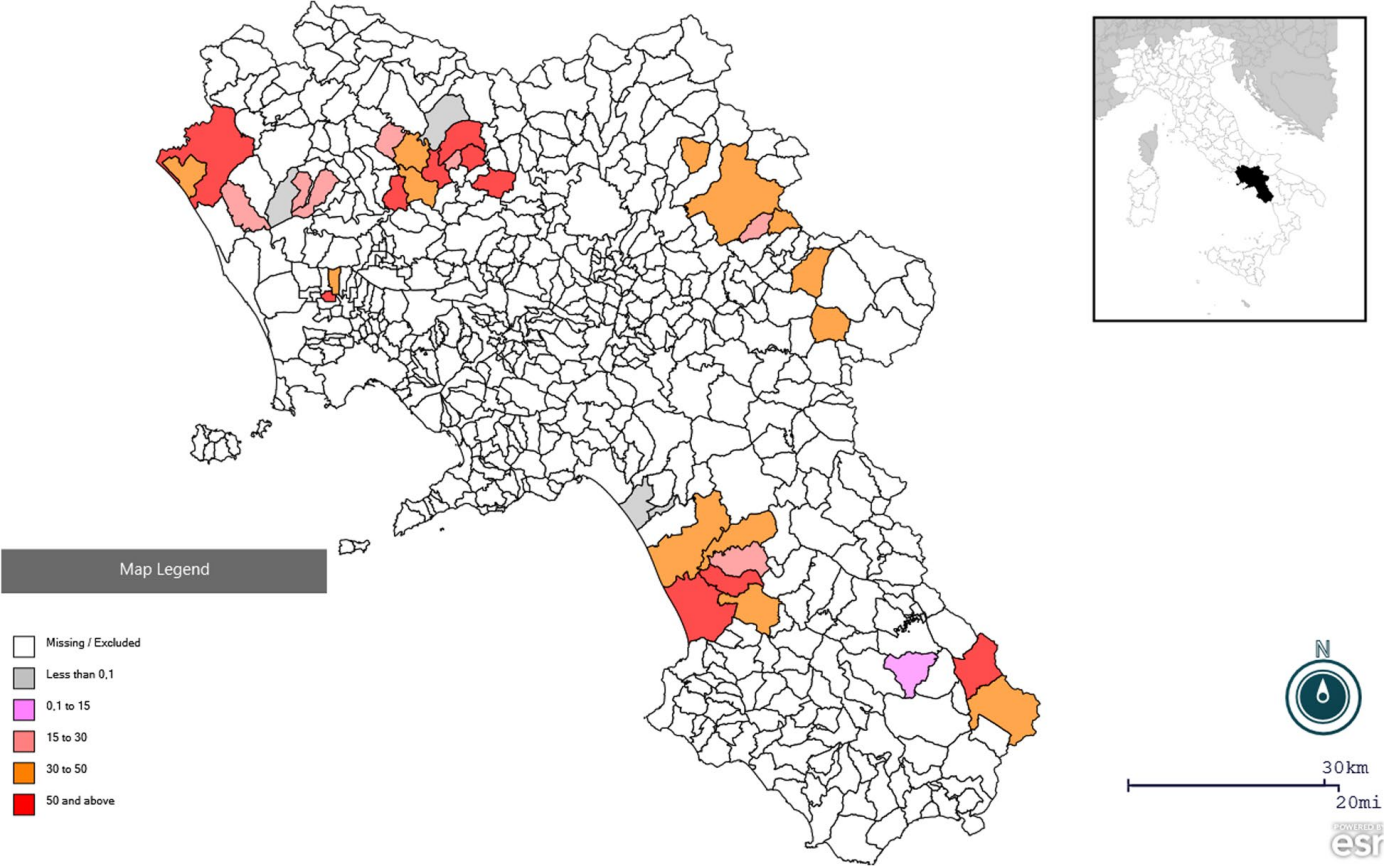
Spatial distribution of positive herds for Schmallenberg virus (SBV) antibodies in Campania region, southern Italy. A large proportion of the sampled districts had seroprevalence between 30% and 50% or higher. The map was created using the geographic information system Epinfo

Eight hundred twelve samples belonging to 52 farms (where SBV vaccines were not used and whose descriptive information is summarized in Supplementary Data 1) were tested for the presence of antibodies against SBV using a commercial enzyme-linked immunosorbent assay (ELISA). Our results showed an overall seroprevalence of 40.5% at the individual level (38.2% in cattle and 43% in water buffalo), whereas a prevalence of 90.4% was observed at the herd level (47 out of 52 herds showed at least one seropositive animal) (Table 1). In particular, only one out of twenty-two cattle herds and four out of thirty buffalo herds were negative. A very high seroprevalence was observed in all the provinces (Fig. 1), with no statistical difference between them, although higher values were observed in Benevento (48.5%). High seropositivity was also combined with high antibody titers. Positive samples were further tested using a virus neutralization test (VNT), to quantify antibody titers and determine the specificity of antibodies detected by ELISA. The distribution of antibody titers is provided in Table 2 and revealed that 22% (182/812) of the animals showed a titer between 1:128 and 1:256. As 17 ELISA-positive samples were negative in VNT, a triplex ELISA assay was additionally used to exclude possible cross-reaction with other bunyaviruses, specifically Akabane and Shuni virus. All samples showed antibodies against the glycoprotein C (Gc) of SBV and were negative for the other Simbu virus except for one sample that was negative for Gc antibodies of all three viruses.

**Table 1:** Univariate analysis (chi-square) of individual potential risk factors (species, age, and origin) for Schmallenberg virus seropositivity

Factor	n	Positive	%	95%CI	χ^2^	*p*-value
**Total**	812	329	40.5	37.1–43.9		
**Species**						
** Cattle**	424	162	38.2	38.2–47.6		
					1.96	0.161
** Buffalo**	388	167	43	38.1–48		
**Age**						
** ≤ 24 months**	311	139	44.7	39.2–50.2		
					3.65	0.056
** > 24 months**	501	190	37.9	33.7–42.2		
**Origin**						
** Born in the farm**	436	174	39.9	35.3–44.5		
					0.145	0.703
** Purchased**	376	155	41.2	36.2–46.2

**Table 2:** Antibody titers obtained using virus neutralization assay (VNT) on ELISA-positive animals

	***n***						
Antibody titer	**1:4**	**1:8**	**1:16**	**1:32**	**1:64**	**1:128**	**1:256**
**Species**							
Cattle	3	7	7	21	33	30	61
Water buffalo	4	3	14	26	29	59	32
Total	7	10	21	47	62	89	93

Univariate analysis revealed the absence of correlation between individual factors (species, age, and origin) and SBV positivity (Table 1), although water buffalo, younger and purchased animals revealed higher seroprevalence. There was a higher seroprevalence, even if not statistically significant, depending on the type of herd (fully stall fed or partly grazed) (Table 3). Regarding the environmental factors, altitude, mean annual temperature, and distance from the coast were associated with higher seroprevalences. Seroprevalence did not differ according to annual rainfall of the location (Table 3).

**Table 3:** Univariate analysis (chi-square) of herd and environmental potential risk factors (location, type of herd, presence of other ruminants, farm dimension, annual mean temperature, altitude, annual rainwater, distance from the coast) for Schmallenberg virus seropositivity

Factor	n	Positive	%	95%CI	χ^2^	*p*-value
**Total**	812	329	40.5	37.1–43.9		
**Location**						
** Avellino**	114	43	37.7	28.8–46.6		
** Benevento**	99	48	48.5	38.6–58.3	3.77	0.299
** Caserta**	298	114	38.2	32.7–43.8		
** Salerno**	301	124	41.2	35.6–46.8		
**Type**						
** Fully stallfed**	695	290	41.7	38.1–45.4		
					2.9	0.087
** Partly grazed**	117	39	33.3	24.8–41.9		
**Presence of other ruminants**						
** Yes**	211	86	40.8	34.1–47.4		
					0.007	0.93
** No**	601	243	40.4	36.5–44.7		
**Farm dimension (number heifers)**						
** < 100**	202	73	36.1	29.5–42.8		
					2.14	0.114
** > 100**	610	256	41.2	38-45.9		
**Mean annual t°**						
** ≤ 16 °C**	424	150	35.3	30.8–39.9		
					9.72	0.002
** > 16 °C**	388	179	46.1	41.2–51.1		
**Altitude (a.s.l.)**						
** ≤ 250 m**	483	211	43.7	39.3–48.1		
					4.96	0.026
** > 250 m**	329	118	35.9	30.7–41		
**Annual rainwater (cm)**						
** ≤ 1000**	214	86	40.2	33.6–46.8		
					0.013	0.909
** > 1000**	598	243	40.6	36.7–44.6		
**Distance from the coast**						
** ≤ 20 km**	302	140	46.4	40.7–52		
					6.8	0.009
** > 20 km**	510	189	37.1	32.9–41.2		

All the variables showing a *p* value < 0.2 were included in the multivariate logistic regression. All variables were included, since they met the criteria for multicollinearity (VIF always less than 2.5). The results, shown in Table 4, revealed a correlation between SBV positivity and mean annual temperature and age. The odds ratio was 1.34 times higher for testing positive for SBV antibodies in young animals than in older animals. Moreover, the odds ratio of positive tests was 1.4 times higher in animals raised in districts with a mean annual temperature above 16 °C. Other variables showed non-significant results in multivariate analysis.

In addition to the serological analyses, an SBV-specific real-time RT- PCR was carried out on 125 individual sera selected as described in the Methods section. Overall, 6 out of the 125 serum samples (4.8%) were positive. The molecular positivities (four seronegative animals and two seropositive animals) were all found in two provinces (Caserta and Salerno) and belonging to farms that had a consistent viral circulation (herd seroprevalence greater than 60%). The small number of PCR-positive animals precluded any statistical analysis. In general, positive samples had high quantification cycle values, which prevented any further study, such as full or partial genome sequencing.

## Discussion

This study represents the first survey of SBV infection in southern Italy. The climatic conditions in southern Italy are extremely favorable for the viral-vector life cycle [25]. Consequently, relatively high seroprevalences against SBV could be detected in mammalian hosts. The individual and herd prevalence were consistent with the prevalence observed in other European countries. For example, in Ireland an individual seroprevalence of about 35% and a herd prevalence of 50% were recorded during two years of surveillance (2012-13) [26]. In 2012, a comprehensive study conducted in the Netherlands on 4439 dairy cattle revealed a seroprevalence of 63.4% [27]. In Poland, a 6-year serosurvey found a seroprevalence of 47% [28]. Regarding the Mediterranean basin, a cross-sectional study made in Turkey found a seroprevalence of about 29% among ruminants, with cattle revealing the highest seroprevalence (40.7%) and the lowest molecular positive rate (only 1%), while small ruminants showed lower seroprevalence and a 5% PCR-positive rate [29]. This trend has been confirmed also in Kosovo in a recent work [30]. In Spain, the seroprevalence detected during the outbreaks recorded in 2012 reached 70% [31]. Interestingly, this prevalence was observed in countries where the clinical impact of the disease was detected in several species. In the Campania region no clinical outcomes have been described and neither outbreak was recorded nowadays.

For the first time, we assessed the seroprevalence of SBV in Mediterranean water buffalo, and, for the first time, assessed the role of water buffalo in the epidemiology of SBV. Our findings revealed that this species, along with other ruminants, should be considered one of the main hosts of SBV. Other surveys involving this species across the world have been described in China in a preliminary study, where 4 out of 21 water buffaloes tested positive for the presence of antibodies using an SBV ELISA that allows for the detection of multiple Simbu serogroup viruses [32]. In Turkey, only two of the 130 sampled animals tested positive, reaching a seroprevalence of 1.5% in Anatolian water buffalo [29]. For strictly ethological considerations, the water buffalo is a suitable host for the transmission of viruses transmitted by *Culicoides*, as it prefers wet, swampy environments near water sources (in fact, the latter are often used as environmental enrichment, which is beneficial for animal welfare) [33]. These characteristics correspond to optimal conditions for *Culicoides* oviposition, and it is remarkable that no surveillance efforts have been directed at this species in the past.

The specificity of antibodies against SBV was also confirmed by VNT. A high percentage of animals showed high antibody titers in VNT (included between 1:128 and 256) suggesting recent exposure. This is corroborated by molecular positive animals that also demonstrated how the vector season in European countries is becoming increasingly prolonged as a result of global warming. But a total of 25 samples were negative in the VNT (antibody titers less than or equal to 1:8), raising doubts about the positivity for other bunyaviruses described in the Mediterranean regions, especially since the nucleocapsid protein-based ELISA reacts not only with anti-SBV antibodies but also with antibodies against closely related viruses of the Simbu serogroup [34, 35]. In other Mediterranean countries, the circulation of the simbuviruses Akabane virus (Turkey and Israel) and Shuni virus (Israel) have been reported and, therefore, we tested also the Italian samples for antibodies against these viruses [36, 37]. However, the samples were negative for Akabane and Shuni virus antibodies and were confirmed as SBV positive (except for one that was considered as false positive in the first commercial ELISA).

It is essential to provide plausible answers to the following questions: Why did we find such high seroprevalences although outbreaks of clinical cases have not been reported from the region? Is what we observed the consequence of old outbreaks that went completely unnoticed (or were perhaps mistaken for bluetongue disease), or is it a wake-up call for an imminent outbreak? Seroprevalences of this kind should be considered normal if we think of the seroprevalences observed in post-epidemic conditions. Immunity to this infection is quite long lasting, with 80% of adult dairy cows still having detectable antibodies to SBV at least 24 months after the virus was estimated to enter the herd [38–40]. However young animals lose passive immunity between 5 and 6 months, making them susceptible [38–41]. A recent study found higher prevalence in the month of October, which coincides with the end of the vector season [29]. In Belgium, peaks of seropositivity were detected between February and April [42]. The lack of historical data makes it difficult to understand the current situation, but the prevalences obtained are comparable to those obtained in European studies demonstrating recirculation of the pathogen. Furthermore, outbreaks have been identified in some countries between 2020 and today (Albania, Germany, Turkey as regards the detection of antibodies but also Spain with regard to the detection of RNA in *Culicoides* biting midges, and Denmark with regard to abortigenic events related to SBV infection) [14, 20, 29, 30]. Recent works have anticipated that SBV will re-emerge in future, and hence, it represents a constant threat for ruminant populations [8, 42, 43]. A significant epidemic with clinical cases following re-emergence every three to four years is possible as long as SBV is present in Europe [6]. Re-emergences in Germany were described in 2014 and 2019, three and eight years after the first outbreak [12, 44]. The frequent re-emergence of SBV in Central Europe is the result of declining herd immunity driven by the replacement of animals by seronegative younger stock. The overall decline in herd seroprevalence may have an impact on the spread of viruses and the number of fetal malformation cases due to the infection of susceptible heifers [44].

The lack of reports of abortifacient and teratogenic events linked to Schmallenberg disease outbreaks in southern Italy raises additional concerns. The only reports of the disease remain from the first outbreak in northern Italy. Were such events confounding for bluetongue disease in the rest of Italy? Unfortunately, we are unable to address this question at the moment, and we can only recommend further research to focus on the Italian epidemiological situation of SBV from a genomic point of view.

Our study identified mean annual temperature and age as risk factors for SBV seropositivity. Other recent studies identified environmental factors that correlated with a higher risk of exposure to SBV, such as altitude in Spain [45]. A Portuguese study identified altitude as a possible risk factor [46]. Only in the univariate analysis did it appear to be substantially connected to increased seropositivity in our research. The tendency we are seeing, similar to that found with bluetongue virus, is that the virus reaches high elevations because, due to global warming, the vector is able to carry out its biological cycle, albeit to a limited extent, even at high altitudes [47].

On the other hand, other studies found the opposite outcome regarding the age with older animals with higher seroprevalence than young [48]. Research done in the Netherlands found no significant variation in the seroprevalence of SBV between age groups [27]. This meant that all age groups were equally vulnerable. The existence of maternal antibodies up to 6 months is one of the causes that might explain a higher incidence in young individuals [41].

While antibodies have high durability, the virus in the blood does not, it is detectable for only three to six days. This makes virus detection in serum extremely difficult; in fact, in our study, only 6 samples were positive, with relatively high quantification cycle values, preventing further investigation. The detection of active infection supports the assumption that the high titres of neutralizing antibodies are due to recent infection.

## Conclusions

The current study is the first in southern Italy to provide data on the serological status of SBV in cattle. Furthermore, it represents the first large-scale epidemiological investigation conducted in the water buffalo population. Environmental variables appeared to be implicated in the transmission of this infection; in both univariate and multivariate analyses, greater mean annual temperatures were significantly related with increased seroprevalence. We recommend continued surveillance of SBV to detect fluctuations in virus circulation, as well as additional studies based on virus isolation/characterization and vector surveillance.

## Methods

### Study area and sampling

The current research was carried out in Campania (410000000 N-143000000 E), a region in southern Italy with a land area of 1,359,000 ha overlooking the Mediterranean Sea (a coastline of 350 km). In this area around 165,000 cattle and 300,000 water buffalo were bred (Banca Dati Nazionale dell’ Anagrafe Zootecnica, https://www.vetinfo.it/j6_statistiche/, accessed on 1 October 2020). This region represents the first in Italy for number of water buffalo. Given the lack of comparable studies in Italy, we opted to assume a 0.5 (i.e., 50%) expected prevalence, an absolute precision of 5%, and confidence interval of 95%. Thrusfield’s formula was used to calculate the sample size, which was as follows:

$$\mathrm n\;=\;\mathrm Z\;2\;\times\;\mathrm P\left(1\;-\;\mathrm P\right)/\mathrm d\;2$$

Sampling started in October 2020 and lasted two months, coinciding with blood collection by state veterinarians for the national brucellosis eradication program with approval by the Institutional Ethics Committee of Department of Veterinary Medicine and Animal production (Centro Servizi Veterinari), University of Naples, Federico II (PG/2022/0093419 20 July 2022) [49, 50]. This period coincides in Italy with the last months of the vector season. In this way, we ensure the possibility of identifying numerous seropositives without precluding the possibility of identifying molecular positives.

The study area in 35 different districts resulted in a total of 22 cattle farms and 30 water buffalo farms distributed in four provinces. Only farms where SBV vaccines were not used were chosen, and 424 samples from dairy cows and 388 samples from water buffalo were randomly collected and classified according to the information collected with a questionnaire. Altitude was calculated using the geographic coordinates of the farms. The Italian National Meteorological Service provided climatic factors collected by weather stations near the sampling farms (http://centrofunzionale.regione.campania.it/#/pages/dashboard).

### Serological assays

Blood samples were taken from the caudal vein with a vacutainer and centrifuged for 10 min at 1000 g to separate sera, which were then harvested and stored at -20 °C until testing. For the PCR assay, an aliquot of each serum sample was stored at -80 °C. IDEXX Schmallenberg Ab Test was used to test each serum sample for SBV specific IgG antibodies according to the manufacturer’s instructions (IDEXX Laboratories). This test is a recombinant nucleoprotein-coated ELISA that has been validated in several ruminant species (including bovine). Because of its good diagnostic performance, it is widely used for routine diagnostics and large-scale epidemiological investigations. Positive samples were further tested using a virus neutralization test (VNT), which was performed with minor modifications according to Pejakovic et al. methodology [51]. Baby hamster cells (BHK-21) were cultured in Dulbecco’s Modified Eagle Medium (DMEM) (Corning) supplemented with 10% fetal bovine serum and Normocin (Invivogen). BHK-21 (4 × 10^4^ cells in 150 µl/well total) was seeded on 96-well plates and grown to 80–90% confluency. To obtain a 1:4 starting dilution in DMEM, the first wells of a dilution plate were filled with inactivated serum (for 30 min at 56 °C). A viral isolate of SBV (BH80/11–4 provided by Friedrich-Loeffler-Institut and Istituto Zooprofilattico Sperimentale dell’Abruzzo e del Molise “G. Caporale”) was given to the wells (500 TCID_50_ per well). Serum and virus were pre-incubated overnight at 37 °C before being added to the cells and incubated for 4 days. Sera with a neutralization titer greater than 1:8 (presence of a cytopathic effect) were considered positive. The samples that tested positive by ELISA but negative by VNT were further processed by using an in-house triplex-ELISA assay to exclude any cross reaction with Akabane and Shuni viruses [34].

### Real-time RT-PCR

RNA was extracted from blood serum using a commercial extraction kit (QIAamp viral RNA Mini Kit, Qiagen) following the manufacturer’s instructions. Based on the state of conservation (discarding hemolytic samples) and the serology result, a total of 125 serum samples was chosen to be tested in real-time RT-PCR. Individual samples were chosen based on the results of ELISA and VNT (samples belonging to highly positive herds that resulted in seronegative or seropositive with low antibody titers were selected). The total of 125 samples included 45 samples showing a low antibody titer (resulted positive in ELISA and with titer lower than 1:32 in VNT) and 80 seronegative samples. All the RNA samples were retrotranscribed using a commercial kit (iScript™ cDNA Synthesis Kit, BioRad). For the reaction mix (20 µL), 2 µL of template, 2× SYBR Green I master mix, and a final concentration of 500 nM of each primer (forward primer 5’ CAGGATGTCAGGATATCTAG 3’ and reverse primer 5’ TCCCTTAACCTCAGCAA 3’) were used [52]. A reference strain was used as positive control (SBV-BH80/11–4). The following profile was used: 10 min at 95 °C, followed by 40 cycles of 10 s at 95 °C, 10 s at 56 °C, and 10 s at 72 °C. Temperature profiles and melting curve analysis were evaluated using CFX96™ Real-Time PCR Detection System (Biorad).

### Risk factor analysis

The animal-level prevalence and the information concerning sampled animals were used for the risk factor analysis. Bivariate analyses of potential risk factors for SBV seropositivity at the animal level were performed using the chi-square test. The serological result (positivity or negativity) was considered the dependent variable, whereas the information obtained with the epidemiological questionnaires represented the independent variable. The independent variables used were age, species, province, origin, type of stabling, dimension of herd, coexistence with other ruminants, altitude, mean annual temperature, total annual rainfall, distance from the coast. Chi-square statistics were used to examine the relationship between dependent and independent variables. A *p*-value less than 0.05 was considered significant, and < 0.2 value was used as a criterion for selecting the variables to be included in the multivariate logistic regression. Using the forward elimination approach, a multivariable logistic regression analysis of putative risk factors for SBV seropositivity was performed on variables having a *p*-value of 0.20 in the univariable logistic regression analysis. Odds ratio (OR) and 95% confidence intervals were used to assess the degree of relationship between independent factors and SBV seropositivity. Fit models were compared using the Akaike Information Criterion (AIC) and selected as those that best fit the data. Collinearity was checked using Variance inflation factor (VIF). MedCalc Statistical Software version 16.4.3 (MedCalc Software, Ostend, Belgium; www.medcalc.org) and JMP version 14.1.0 were used for statistical analysis (SAS Institute Inc.). A map illustrating the spatial distribution of positive districts was created using the geographic information system Ep Info (https://www.cdc.gov/epiinfo).

**Table 4:** Logistic regression model for the association of potential risk factors (*p* < 0.2) with Schmallenberg virus positivity

Factor	Coefficient (ß)	Standard error	OR	OR CI%	*p*-value
**Mean annual temperature (> 16 °C)**	0.34	0.152	1.4	1.04–1.9	0.026
**Age (≤ 24 months)**	0.297	0.148	1.34	1.01–1.8	0.045
**Distance from coast (< 20 km)**	-0.291	0.157	1.25	1-1.52	0.064

## Supplementary Information

**Additional file 1.**

## Data Availability

All data generated or analysed during this study are included in this published article (and its supplementary information files).

## Abbreviations

SBV: Schmallenberg virus
EFSA: European Food Safety Authority
ELISA: Enzyme Linked Immunosorbent Assay
VNT: Virus Neutralization Test
Gc: Glycoprotein C
DMEM: Dulbecco’s Modified Eagle Medium
BHK-21: Baby hamster kidney cell
OR: Odds Ratio
AIC: Akaike Information Criterion
VIF: Variance inflation factor
